# Promise and Pitfalls of Mitochondrial Replacement for Prevention and Cure of Heritable Neurodegenerative Diseases Caused by Deleterious Mutations in Mitochondrial DNA

**DOI:** 10.3389/fncel.2016.00219

**Published:** 2016-09-23

**Authors:** Ananta Paine, Manoj Kumar Jaiswal

**Affiliations:** ^1^Division of Allergy/Immunology and Rheumatology, University of Rochester Medical CenterRochester, NY, USA; ^2^Molecular Imaging and Neuropathology Division, New York State Psychiatry Institute, Columbia UniversityNew York, NY, USA; ^3^Department of Psychiatry, Columbia UniversityNew York, NY, USA

**Keywords:** neurodegenerative disease, mitochondria, mitochondrial gene transfer (MGT), mitochondrial replacement techniques (MRT), mitochondrial DNA (mtDNA), mitotherapy

Mitochondria are cytoplasmic organelles present in eukaryotic cells that serve as major source of cellular energy produced through oxidative phosphorylation and thus also known as the power plants of eukaryotic cells. A distinct feature of mitochondria is its capacity to regenerate owing to own set of genomic material containing 37 genes. It is now well-known that mutations in mitochondrial DNA can cause many inherited diseases including ones that affects neurons and nervous systems. Importantly, in contrast to other cells, neuron rely heavily upon mitochondria due to their inability to derive sufficient energy though glycolysis (Wallace et al., 2010). As a results mitochondrial dysfunction severely affects neuronal cells and proved to be central in the pathogenesis of many neurodegenerative diseases such as Amyotrophic lateral sclerosis (ALS), Parkinson's disease (PD), Huntington's disease (HD), Alzheimer's disease (AD) and many others (Johri and Beal, 2012). According to Friedrich Max Müller, Mitochondria's are “Advaya” (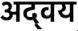) which in Sanskrit means unique and in Upanishads it means ultimate (Max Müller, 1823–1990). Mitochondria are cell's energy factories, divide, multiply, manufacture ATP to fuel all of life's activities. Mitochondrial DNA replacement has been successful in mice and primates and with the refinement of MRT, we hope that it becomes a reality in human. There are more than ~700 known disease-associated mitochondrial DNA (mtDNA) mutations (mitomap.org). Up to 4000 children per year in the US are born with inherited mtDNA disorders (Schaefer et al., 2008).

In recent years, major advances in the field of nuclear transfer techniques offer the possibility to transfer the nuclear material from one cells with damaged and dysfunctional mitochondria to into another cell only containing cytoplasmic material resulted from careful removal of the nuclear material before the transfer. Such transfer gives rise to cells where damaged and dysfunctional mitochondria gets replaced by healthy mitochondria from the donor cells keeping the nuclear genomic material unaltered (Falk et al., 2016). This is now known as mitochondrial replacement and the associated techniques are known as mitochondrial replacement techniques (MRT). Mitochondrial gene replacement in oocytes leads to complete replacement of entire mtDNA, applicable to any mtDNA mutation type and eliminates entire spectrum of mtDNA disease. Since genetic corrections will be heritable and passed on to later generations, MRT prevents the need for repeated therapy generation after generation. This significant progress raises the hope that replacement of affected mitochondria in patient's cells can provide curative measures for several mitochondrial diseases. In spite of various ethical and technical concerns, clinicians see huge potential of MRT for cures of several devastating mitochondrial diseases. In this opinion article we highlights an update on new advances and implications of mitochondrial therapy in neurodegenerative disorders and provide insights into studies, suggesting limitations of this advance technology and its future use in clinics. Our hope is that this article will provide a platform for further critical discussion of this pertinent issue.

## Mitochondrial dysfunction and neurodegenerative diseases

Neurological diseases are heterogeneous in nature and affect millions of people around the world. Genetic association studies pinpointed ~150 genetic diseases in which abnormalities in a gene encoding a protein involve in regulation of mitochondria (Calvo et al., 2016). For example, abnormalities in mitochondrial functions have been attributed to be either a root cause or a major driving mechanism involved in ALS disease (Jaiswal, 2013, 2014). In ALS, superoxide dismutase 1 (SOD1) has been proven to be a key gene involved in the disease pathogenesis (Jaiswal, 2012). SOD1 has been found to be mutated in at least in ~20% familial ALS (fALS) cases. Most importantly, studies over the years have identified mitochondrial dysfunction and associated oxidative stress, reactive oxygen species (ROS) formation and calcium dysregulation to be the critical effecter mechanism through which mutated SOD1 affects neuronal function and survival (Jaiswal and Keller, 2009; Jaiswal et al., 2009; Grosskreutz et al., 2010). Similarly, in case of PD, scientists have focused on the function of Parkin and PTEN-induced putative kinase 1 (PINK1), two proteins mutated in familial, early-onset Parkinson's disease and recommended drugs for therapies that boost mitophagy or stimulate activity of Parkin/PINK1 (Narendra and Youle, 2011). Recently it was shown that in autosomal dominant HD where mhuntingtin (mHTT) mutated protein localizes to the outer mitochondrial membrane, where it wields harmful effects on mitochondria by diminishing mitochondrial motility, alters mitochondrial morphology, fusion and fission, causes calcium dysregulation, reduces oxidative phosphorylation, and depolarizes the mitochondrial membrane potential in HD patients (Reddy et al., 2009). Some of the therapeutic approaches recently tested to target mitochondrial fission protein to which mHTT binds, in mice and cells from humans with HD not only curtailed the toxicity of HTT, but also restored normal motor function in symptomatic HD mice (Song et al., 2011; Di Pardo et al., 2012). Douglas Wallace discovered that mutation in one of the mitochondrial tRNA genes precedes the pathological change, which leads to mitochondrial dysfunction, are the hallmarks of AD (Coskun et al., 2010). So, in general, dysfunctional mitochondria are the hallmarks in the major neurodegenerative diseases.

## Mitochondrial dynamics, turn over and its role in neurodegenerative diseases

Mitochondria have turn over with a half-life of ~25 days in neurons (Menzies and Gold, 1971). Neurons, like other cells critically depend upon mitochondrial functions for long-distance transport of mitochondria to the synapse, isolation and removal of faulty mitochondria from synaptic sites and metabolic demands that require high bioenergetic outputs and often associated with enhanced production of ROS. Continuous buildup of ROS leads to oxidative damage and impaired protein homeostasis within mitochondrial micro-domains (Lin and Beal, 2006; Jaiswal, 2013, 2014). Therefore, mitochondria have internal “mitochondrial quality control (MQC)” machinery to maintain a vigorously functional, and healthy mitochondrial number and mtDNA mutations by versatile proteolytic enzymes and exchange of components during fusion/fission to maintain normal function (Rugarli and Langer, 2012). Asymmetric fission mechanism segregate damaged components into one daughter mitochondrion, which is removed by autophagy (Twig et al., 2008). Moreover, during fusion/fission mitochondria form a tubular network via a dynamic process and dictated by the fine balance between mitochondrial fusion and fission. Altered balance of mitochondrial dynamics leads to pathogenesis of complex neurodegenerative disorders such ALS, AD, PD, and HD. For details see Burté et al. (2015). Selective mitochondrial mitophagy removes damaged mitochondria and closely associated to biogenesis, which permits replacement of mitochondria and assembly of multiple mitochondrial proteins (Kim and Lemasters, 2011). Thus, maintenance of protein homeostasis through MQC is one of the key aspects in preservation of functional integrity of neuronal mitochondria (Bohovych et al., 2016). MQC involved in removal of impaired misfolded proteins and regulation of assembly, maturation and cleavage of proteins.

## Mitochondrial resuscitation/replacement in neurodegenerative disorders

**Figure 1:**
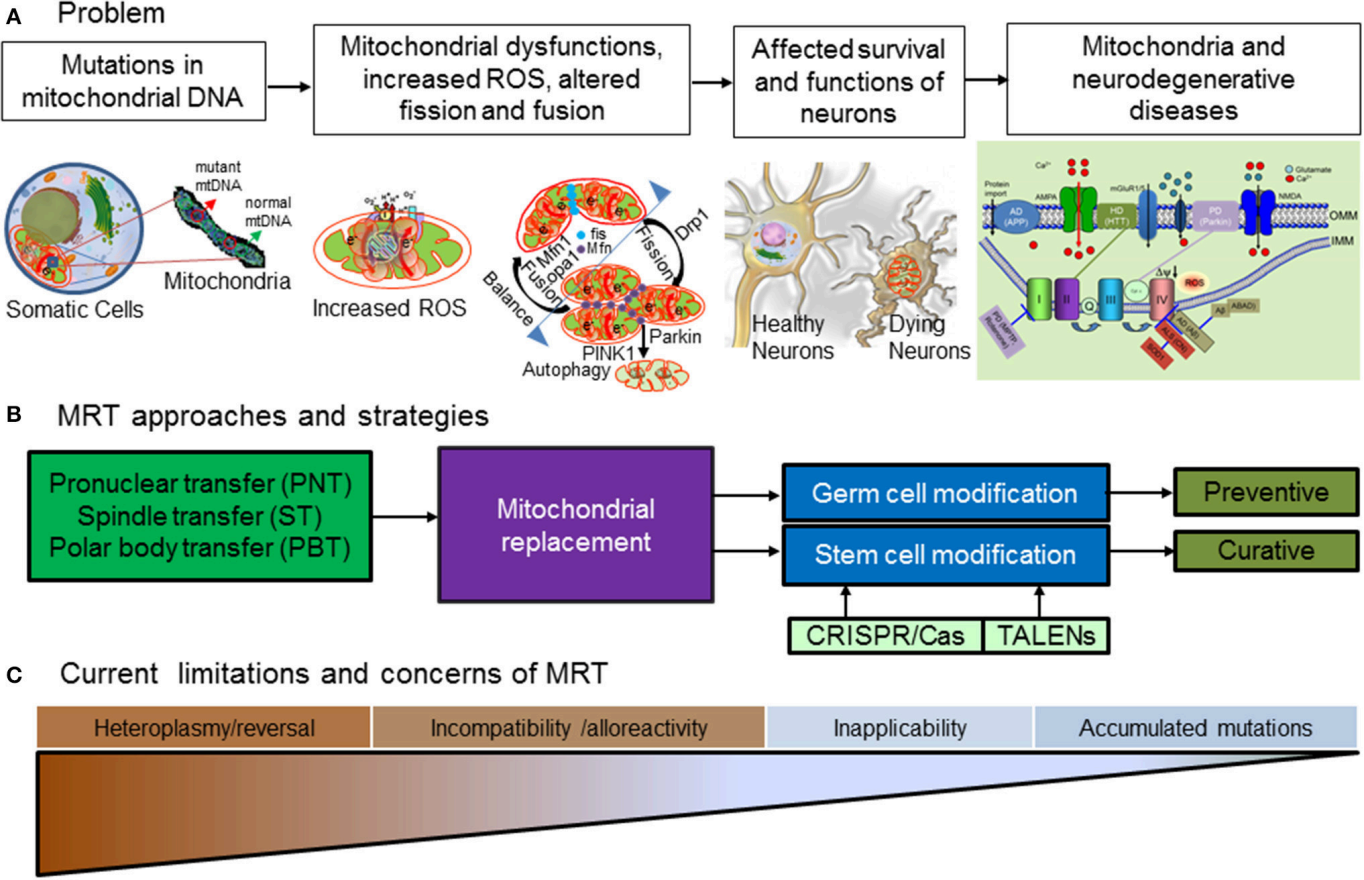
**Mitochondrial replacement for neurodegenerative diseases. (A)** Problems associated with mutations in mitochondrial DNA leads to generation of toxic ROS which impair calcium homeostasis. Oxidative stress build up, in turns impair balance between fusion and fission of mitochondria accelerating vicious cycle of mitochondrial damage ultimately affecting survival and function of neurons and results in cell death leading to many neurodegenerative diseases. **(B)** MRT currently involves different strategies such as pronuclear transfer (PNT), spindle transfer (ST) **(C)** polar body transfer and other related techniques for mitochondrial modifications. Pronuclei (PN) are membrane-enclosed entities in the zygote of mammals that house the male and female chromosomal contributions. In PNT, pronuclei are removed from single-cell stage embryo at day one and transferred to a second embryo created using a donor egg with healthy mitochondria. This tailored embryo can develop and transferred into mother. In ST technique, first spindle from donor and mtDNA-carrying unfertilized oocytes are extracted into karyoplasts and thereafter karyoplasts from patient oocytes are fused to donor cytoplasts and fertilized with the partner's sperm to create mtDNA-mutation free embryos. During meiosis, mammalian oocytes undergoes two reductive divisions with uneven cytoplasmic segregration giving rise to two small cells containing DNA known as polar bodies (PB)s which eventually disintegrates. The first PB (PB1) contains a diploid set of chromosome whereby the second polar body (PB2) contains a haploid set. In PBT techniques, transfer of PB1 and PB2 into appropriate oocyte or zygotic cytoplasm supports the normal completion of meiosis giving rise to the viable offspring after completion of its full term development. Advance genomics tools such as Transcription Activators like effector nucleases (TALENs), and CRISPR/Cas edited iPS cells with corrected mutations in mitochondrial DNA possibly can overcome some of technical challenges MRT facing right now. MRT can be achieved by germ cell (preventive) and stem cell (curative) modifications. **(C)** Major challenges of MRT are: (a) heteroplasmy and reversal, (b) incompatibility and alloreactivity, (c) inapplicability of some techniques in human cells, and (d) accumulated mutations in mitochondria.

Although many new candidate drugs or treatment strategies are well on the way, unfortunately the fact remains that there is still not a single broadly applicable effective cure available for ALS, MS, PD, HD, and AD. Moreover, those suffering from familial neurodegenerative diseases and carries the harmful mutations in their nuclear and mitochondrial genetic materials face significant risk of passing over these fatal diseases to their children. Thus, many individuals with genetic predisposition and mutated mtDNA combined with other contributing factors are likely to be affected ruining their life without any hope for cure. Therefore, there are eminent needs to find cure for such neurodegenerative diseases. While approaches such as gene therapy or other techniques will be needed for correcting the harmful errors in nuclear genome, recent advancements now provides the opportunity to correct the errors in the mitochondrial genetic material applying different versions of recently developed MRT procedures. We can think of two different modes of applying the related therapeutic approaches, (a) preventive and (b) curative (Figure 1).

In case of different neurodegenerative diseases with known mutation in mtDNA, corrective measures such as mitochondrial replacement by *in vitro* fertilization can serve as preventive cure.

As a preventive measure, MRT can be applied to replace the dysfunctional mitochondria in the germ line cells thereby avoiding harmful presence of the faulty mitochondrial genes in the children. This can be achieved by harvesting and transferring the nucleus of a woman carrying mtDNA mutations to the eggs of a woman donor with healthy mtDNA to be fertilized. This allows the “original” mother to avoid transmitting faulty mitochondrial genes to the fetus, which inherit all mtDNA from the egg. An alternative method involves fertilizing eggs from both women *in-vitro*. Then, physicians remove and transfer chromosomes from the nucleus of one egg to the egg from the woman with healthy mitochondria. This technique is dubbed by the media as “three-parent *in-vitro*-fertilization.”

Currently, three established techniques for mitochondrial replacement exist for germ line therapies to circumvent mtDNA based disease transmission: (1) pronuclear transfer (PNT, McGrath and Solter, 1983; Craven et al., 2010), (2) spindle transfer (ST, Tachibana et al., 2009; Cohen and Alikani, 2013; Tachibana et al., 2013), and (3) polar body transfer (PBT; Wang et al., 2014; Wolf et al., 2015). In PNT, pronuclei are removed from single-cell stage embryo at day one and transferred to a second embryo created using a donor egg with healthy mitochondria. In ST technique, first spindle from donor and mtDNA-carrying unfertilized oocytes are extracted into karyoplasts and thereafter karyoplasts from patient oocytes are fused to donor cytoplasts and fertilized with the partner's sperm to create mtDNA-mutation free embryos. Whereas in PBT techniques, first or second polar bodies are transfer into appropriate oocyte or zygotic cytoplasm supports the normal completion of meiosis giving rise to the viable offspring after completion of its full term development.

These techniques have distinct advantages and disadvantages. In the recent years, these techniques have matured and have been tested in various model organisms with some degree of success. Recent PNT results between zygotes and nuclear spindles among oocytes suggest the possibility of its use in humans (Tachibana et al., 2009; Paull et al., 2013). Apart from the above-mentioned preventive application, mitochondrial replacement can also serve as a curative strategy. For this, MRT can be applied to manipulate harvested stem cells collected from patients and thereafter doing the homologous stem cell transplantation after correcting the mitochondrial defects. It is important to mention that recently scientist successfully demonstrated that genetically corrected induced pluripotent stem cells (iPSCs) can be created from fibroblasts from patients with mitochondrial mutations (Ma et al., 2015). A similar approach has been tested earlier on macaques produced normal offspring (Tachibana et al., 2009). These discoveries indicate the feasibility of such techniques for future therapeutic approaches.

## Technical challenges, concerns and recent advancements

While recent developments in the field of MRT raising high hope for cure, there remain major technological challenges and concerns as outlined below.

### Heteroplasmy and reversal

Mitochondria constantly undergo fusion and fission (Karbowski and Youle, 2003; Youle and van der Bliek, 2012). Moreover, during mitochondrial transfer chances of those residual damaged mitochondria remains in the cells can compromise the effectiveness and long-term benefit of the MRT. As a result, a prerequisite for ensuring successful and effective replacement of damaged mitochondria is avoiding or critically minimizing the carryover of damaged mitochondria. Most effective replacements of damage mitochondria are thus needed to circumvent this problem and undesired effect. Prior work already showed that different techniques has different efficacy in minimizing such carryover. While in case of techniques such as ST, first polar body transfer (PBT1) or PNT, chances of carry over and heteroplasmy remains high, in case of second polar body transfer (PBT2) such carry over remains below detection level and ~1% (Yamada et al., 2016). Thus, in future PBT2 or other effective novel techniques might serve as a technique of choice for MRT. A recently published article reported improved PNT technique which solve the problem of mitochondrial carry over and reduce the number of defective mitochondria transferred along the nuclear DNA to the donor cell (Hyslop et al., 2016).

### Incompatibility and alloreactivity

Since the conception of mitochondrial replacement as a strategy, a major concern has been the possible incompatibilities of donor's mitochondria with the nuclear from unrelated individuals. However, there are no strong evidence available strongly supporting this concern especially in case of human. Moreover, current studies indicate persistent compatibility between nuclear genome and donor's mitochondria (Mitalipov and Wolf, 2014). Apart from nuclear-mitochondrial compatibility, alloreactivity also emerges as serious concern if adequate attention not paid to avoid it. This aspect is especially relevant for future curative applications to treat patients with modified stem cells after mitochondrial replacement. However, established approaches such as immune suppression might be able to provide us viable solution to circumvent such undesired side effects if needed. Moreover, inexpensive genetic testing and matched donors also can provide us ways to prevent such mismatches as mean to eliminate or reduce chances for such undesired immune response.

### Inapplicability of certain techniques in human cells

A major concern that effectiveness of many of these associated procedure has been mainly shown successfully in animal studies and applicability of such procedure still remained to validated in human. While future studies will be needed to achieve such validation to confirm the applicability of such techniques, recent studies using human cells shows the initial evidences for the effectiveness of MRT in human (Yamada et al., 2016).

### Accumulated mutations in mitochondria from aged persons

A recent discovery on stem cell mitochondria points that iPS cells clones derived from elderly adults show accumulation of DNA mutations and therefore screening cell lines for mitochondrial mutation is important for clinical use (Kang et al., 2016). Findings came with major conclusions that iPS cells derived from older patients tend to show DNA mutations which can impact metabolic function in iPS cells. However, recent improvement of PNT which solve the problem of mitochondrial carry over reduce the risk of mutant mtDNA (Hyslop et al., 2016). It is also important to mention that currently MRT experts think that physiological behavior of mitochondria is very different in embryonic stem cells compared to normal human development and mutations of iPS mitochondria might not be true for human and therefore procedure is safe.

## Conclusion and future directions

In spite of all of the concerns, it seems MRT has considerable potential for preventing and possibly curing certain forms of neurodegenerative diseases caused due to damaged or dysfunctional mitochondria. Bench-to-bedside implementation of above-mentioned approaches will be based on information from a variety of scientific data and clinical trials of safety and efficacy. Mitochondrial replacement combined with stem cells might provide us future cures at least to a subset of patients where structural and functional mitochondria-derived abnormalities dictate disease pathology and progression. One key factor for success of this technology will be the cost effectiveness and viability in clinical set ups. Technology is still maturing and there are several concerns that need to be addressed regarding the possible differences between the animal models, preclinical studies and human clinical conditions. While this approach can serve as a good alternative approach, it might have limited scope considering the disease heterogeneity and multiple mode of the disease pathogenesis. Nonetheless, even if the techniques can be successfully implemented in few of the well-equipped centers and clinical facilities, that still be extremely beneficial to patients who are currently suffering without hope for a cure. Only long-term studies can tell us for certain if the technique is mature enough to be applied at broad scale in clinics. We also hope there will be a consensus built across our society and political arena on the use of MRT. Furthermore, we hope that the recent and future developments of MRT research will further improve the efficacy and safety of the related techniques and will provide the therapeutic benefit to the affected individuals and to avoid the transmission of such life threatening diseases in their future generations.

## Author contributions

All authors listed, have made substantial, direct and intellectual contribution to the work, and approved it for publication.

## Disclaimer

The views expressed in this article are those of the authors and do not reflect the official policy or position of the University of Rochester Medical Center, or NYSPI/Department of Psychiatry, Columbia University Medical center.

### Conflict of interest statement

The authors declare that the research was conducted in the absence of any commercial or financial relationships that could be construed as a potential conflict of interest.

## Abbreviations

AD: Alzheimer's disease
ALS: amyotrophic lateral sclerosis
CRISPR/Cas: clustered regularly interspaced short palindromic repeats and CRISPR-associated
fALS: familial amyotrophic lateral sclerosis
FDA: food and drug administration
HD: Huntington's disease
HFEA: human fertilization and embryology authority
MRT: mitochondrial replacement technique
mHTT: mhuntingtin
mtDNA: mitochondrial DNA
mSOD1: mutant superoxide dismutase 1
MNs: motor neurons
MND: motor neuron disease
MQC: mitochondrial quality control
PD: Parkinson's disease
PINK1: PTEN-induced putative kinase 1
PBT: polar body transfer
PNT: pronuclear transfer
ROS: reactive oxygen species
ST: spindle transfer
SOD1: superoxide dismutase 1
TALENs: Transcription Activators like effector nucleases
[Ca^2+^]_i_: cytosolic calcium.
